# Phytochemical Screening and Bioactivity of *Ludwigia* spp. in the Control of *Plutella xylostella* (Lepidoptera: Plutellidae)

**DOI:** 10.3390/insects11090596

**Published:** 2020-09-03

**Authors:** Eliana Aparecida Ferreira, Silvana Aparecida de Souza, Alberto Domingues, Matheus Moreno Mareco Da Silva, Isabella Maria Pompeu Monteiro Padial, Emerson Machado de Carvalho, Claudia Andrea Lima Cardoso, Sandra Verza da Silva, Rosilda Mara Mussury

**Affiliations:** 1 Faculty of Biological and Environmental Sciences, Federal University of Grande Dourados, Highway Dourados-Itahum, km 12, Dourados, Mato Grosso do Sul 79804-970, Brazil; lih.ferreira.ivi@gmail.com (E.A.F.); silvanaadesouza@gmail.com (S.A.d.S.); sandraverza@yahoo.com (S.V.d.S.); 2 Faculty of Agricultural Sciences, Federal University of Grande Dourados, Highway Dourados-Itahum, km 12, Dourados, Mato Grosso do Sul 79804-970, Brazil; zoocg@yahoo.com.br (A.D.); matheusmoreno1909@hotmail.com (M.M.M.D.S.); bellapadial@hotmail.com (I.M.P.M.P.); 3 Techno-Science and Innovation Training Center, Federal University of Southern Bahia-UFSB, Itabuna Access Highway, km 39-Ferradas, Itabuna, Bahia 45613204, Brazil; carvalho.em@gmail.com; 4 Center of Studies in Natural Resources of the State University of Mato Grosso do Sul, Highway Dourados-Itahum, km 12, Dourados, Mato Grosso do Sul 79804-970, Brazil; claudia@uems.br

**Keywords:** *Ludwigia tomentosa*, *Ludwigia longifolia*, *Ludwigia sericea*, *Ludwigia nervosa*, diamondback moth

## Abstract

**Simple Summary:**

*Ludwigia* species have several pharmacological applications, but their insecticidal proprieties have not been tested. This research thus aimed to study the effects of aqueous extracts on the biological characteristics of *Plutella xylostella*. We noted that the *L. tomentosa*, *L. longifolia* and *L. sericea* extracts were active. These species showed the best results regarding their ability to control *P. xylostella* populations, due to the presence of substances that inhibit food consumption and interfere in the morphological and physiological transformations of the offspring and adult oviposition.

**Abstract:**

We tested the bioactivity of aqueous extracts of *Ludwigia* spp. (Myrtales: Onagraceae) on the biological cycle of *Plutella xylostella*. We assessed the duration of and viability during the larval, pupal and adult phases, as well as the influence of the extracts on the fecundity and hatching of *P. xylostella* eggs. Subsequently, we phytochemically screened the extracts. The extracts of *L. tomentosa* and *L. longifolia* reduced the pupal weight instead of prolonging the larval stage of *P. xylostella*. The *L. tomentosa* effect caused higher larval mortality and reduced the fecundity and hatching of *P. xylostella* eggs, and *L. sericea* reduced the egg survival. The phenolic compounds—flavonoids, condensed tannins and alkaloids—were more abundant in *L. nervosa*, *L. tomentosa*, *L. sericea* and *L. longifolia*. The *L. tomentosa*, *L. longifolia* and *L. sericea* extracts were bioactive, and these species showed the best results regarding their ability to control *P. xylostella* populations, because these plants produce substances able to inhibit food consumption and interfere with the morphological and physiological transformations of the offspring and the oviposition of adults.

## 1. Introduction

The *Plutella xylostella* (Linnaeus, 1758) (Lepidopera: Plutellidae) microlepidoptera, popularly known as the diamondback moth, is the most economically important pest for the Brassicaceae family. Around four to five billion US dollars are spent on its control worldwide [1]. Due to huge economic damage, chemical control is the best way to contain *P. xylostella* [2]. In some parts of the world, 20 applications of synthetic insecticides have been registered in a single growing season.

The existence of synthetic insecticides with various formulations have increased crop yields, but their extensive use to control *P. xylostella* has resulted in more rigid selection, leading to the development of resistance to more than 95 different insecticides [3].

If incorrectly used, synthetic insecticides can be harmful; they may be problematic for human health, cause ecological problems [4]; contaminate food products; damage the environment (contamination of water, air and soil resources); increase the resistance and favor the recurrence of pests; eliminate nontarget organisms [5,6] and harm natural enemies [7]. Therefore, it is necessary to adopt techniques and tactics that are less harmful but equally efficient for controlling *P. xylostella*.

Extracts of many plant species show proven insecticidal effects, since they contain diverse active compounds that act synergistically, with attractive or repellent characteristics [8,9,10,11,12,13]. Plants create and use their own secondary metabolites as protection against microorganisms, insects and other phytophagous arthropods [14]. Such compounds become natural candidates for discovering new products that can be used to control insects.

Compounds extracted from plants can be incorporated into integrated pest management (IPM) and act as contact insecticides, repellents and food and/or reproduction suppressors [15]. Herb-based products are often described as benign to beneficial arthropods and the ecosystem [16] and are rapidly decomposed after being exposed to the atmosphere [17], reducing the risk of residues remaining in food. Botanical insecticides can be selective [18] and generally do not cause any resistance, as do synthetic insecticides [19,20].

A number of studies have been performed to determine the insecticidal potential of botanical oils and extracts of certain species—Myrtaceae [21], Rubiaceae [22], Anonnaceae [23,24], Meliaceae, Anacardiaceae [24,25], Apocynaceae [26,27], Sapindaceae and Fabaceae [28]—and the oil of Rutaceae [29] on the development and/or oviposition of *P. xylostella* and, consequently, the occurrence of antibiosis and antixenosis.

Some species of the *Ludwigia* genus show promising sources of anticancer phytochemicals [30] and antioxidants [30,31,32], with antibacterial [31,32,33] and antifungal activity [34]. However, no studies have been reported showing the insecticidal potential of this genus. This study therefore tested the hypothesis that species from this genus have insecticidal potential against *P. xylostella*.

Upon analyzing the antibacterial and antifungal actions of *L. decurrens* Walter [34], *L. abyssinica* A. Rich. and *L. leptocarpa* (Nutt.) Hara, they were found to have saponins [31], and *L. adscendens* (L.) H. Hara was found to contain tannins, alkaloids, flavonoids, terpenes, triterpenoids and phenols.

Secondary metabolites such as flavonoids can be responsible for changes in the biological and morphological characteristics of insects [22]. They also act as phagodeterrent substances [35], similar to alkaloids [36] and tannins [37]. However, there is no report of the insecticidal potential of species of the genus *Ludwigia* to date.

Understanding their effects on the lifecycle of *P. xylostella* could improve the knowledge and encourage future investments in research into this plant genus. Our aim was, thus, to test the bioactivity of aqueous extracts of *Ludwigia* species against *P. xylostella*.

## 2. Materials and Methods

Extracts and bioassays were prepared in the Insect-Plant Interaction Laboratory of the Faculty of Biological and Environmental Sciences at the Federal University of Grande Dourados—UFGD, Dourados, MS, Brazil. The compounds were classified at the Center of Studies in Natural Resources of the State University of Mato Grosso do Sul—UEMS in Dourados, MS, Brazil.

### 2.1. Rearing of P. xylostella

Individuals were maintained under constant temperature (25 ± 2 °C), relative humidity (55% ± 5%) and photoperiod (12 h). The pupae were placed in a transparent plastic cage (9-cm-long × 19-cm-wide × 19-cm-high) until the adults emerged, which were fed with a 10% honey solution. Cabbage leaf discs, manually cut to 8 cm in diameter, were placed on a paper filter and used as an oviposition substrate. These discs were replaced daily, and the eggs were transferred to sterile plastic pots measuring 30-cm-long × 15-cm-wide × 12-cm-high. After hatching, the larvae remained in these containers until they reached the pupal stage.

**Figure 1:**
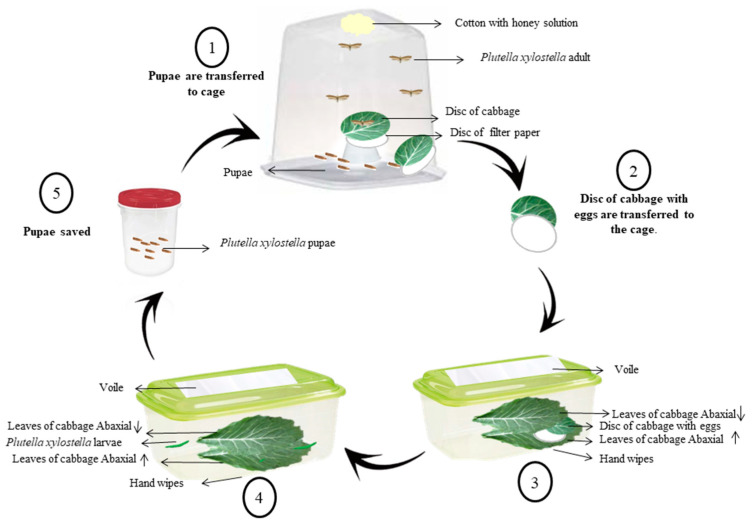
A schematic representation of the methodology used for rearing *Plutella xylostella* by Matias da Silva et al. [39].

The feeding substrate of the larvae was composed of organic cabbage leaves (*B. oleracea* var. acephala) cleaned with 5% sodium hypochlorite solution and later washed in running water. The cabbage leaves were arranged with the adaxial face facing the plastic container and the abaxial face free. The larvae were placed on them, and then, another leaf of cabbage was placed with the abaxial side facing the larvae (Figure 1). This procedure was performed daily, always keeping the leaves higher, and was repeated until the pupae were formed [38].

### 2.2. Botanical Materials

Expanded leaves of *L. tomentosa* (Cambess.) H. Hara, *L. longifolia* (DC.) H. Hara, *L. sericea* (Cambess.) H. Hara and *L. nervosa* (Poir.) H. Hara were collected from highway surroundings in the city of Dourados, MS. The collection area was between the Atlantic Forest and the Cerrado and was beginning to regenerate (secondary succession), with a predominance of herbaceous vegetation (grasses and ferns) and shrub vegetation (22°11′54.92″ S, 54°46′52.15″ O).

The plant species were identified by a specialist in the laboratory of Applied Botany, and the specimens were deposited at the Herbarium of the Federal University of Grande Dourados—UFGD, with the following registration numbers: 6391—*L. tomentosa*, 6389—*L. longifolia*, 6388—*L. sericea* and 6390—*L. nervosa*. The collection of the botanical material was authorized by the Brazilian National Research Council (CNPq)/Council of Genetic Heritage Management (CGEN/MMA), under the number A9ECAC6.

### 2.3. Preparation of Aqueous Extracts

The leaves were dried inside a forced-air circulation oven for three days at a maximum temperature of 40 °C (±1 °C) and then crushed in an industrial mill to obtain a fine powder. Maceration was used to prepare the aqueous extracts. The aqueous extracts were prepared by mixing 5 g of solid plant material with 50mL of distilled water. Next, we used a paper filter to separate the solid material from the extract. The extracts were then refrigerated (10 °C) for 24 h and strained through cheesecloth. Extracts at a concentration (weight/volume) of 10% were obtained.

### 2.4. Bioactivity of Ludwigia spp. Aqueous Extracts against P. xylostella

The methodology for assessing the bioactivity of the plant extracts was based on [22]. The larvae were monitored until they reached the pupal stage (larval duration). The first assessment of mortality was made at 48 h after the confinement of the larvae in the Petri dishes, counting the number of dead individuals and replacing the cabbage leaf discs with others of the same treatment. The following assessments were performed daily, and the leaf discs were changed every 24 h, until the larvae reached the pupal stage or not (larval survival).

The pupae of each treatment were separated in test tubes to assess the time spent in the pupal stage (pupal duration). The pupae were weighed 24 h after pupation (pupal biomass) (Bel Mark Analytical Balance—0.001 g). The duration of this stage was subsequently monitored until the pupae emerged, reaching adulthood or not (pupal survival).

Ten couples from each treatment were separately placed in plastic cages with cabbage leaf discs (diameter of 8 cm) acting as oviposition substrates for assessing the reproductive stage. The cabbage discs were replaced by new ones daily. The discs with the eggs were transferred to Petri dishes to count the number of eggs (fecundity) and to monitor the hatching of larvae with a Motic SMZ-168 series stereoscope.

**Figure 2:**
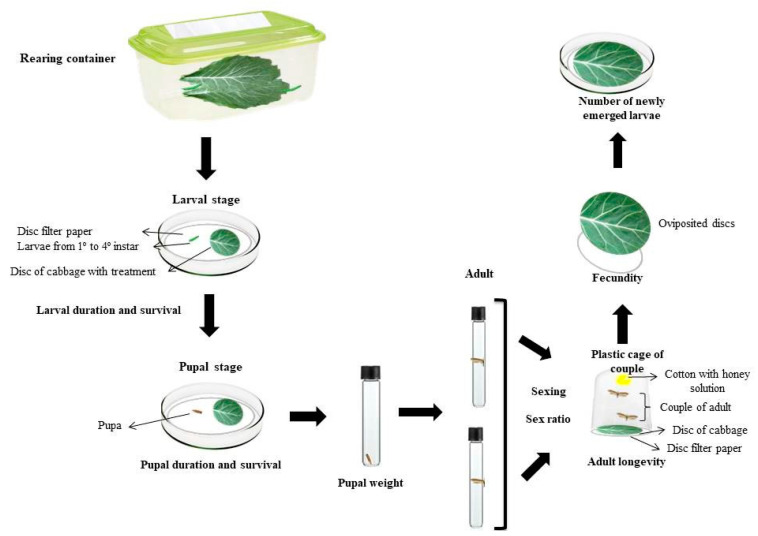
A schematic representation of the methodology used for the evaluation of the biological parameters of *Plutella xylostella*.

The biological parameters assessed were the duration (days) of and survival (in %) during the larval and pupal stages, pupal weight (in mg), longevity of the females and males (days), sex ratio (sr = female/female + male), fecundity (total number of eggs laid throughout life), newly emerged larvae (numbers of eggs hatched in larvae) and survival of the eggs (percentage of eggs hatched in larvae) (Figure 2).

### 2.5. Determination of Polyphenolic Content

The total phenolic content was determined with the Folin–Ciocalteu reagent method [40] and samples at a concentration of 1000 µg/mL. The absorbance was measured using a spectrophotometer (FEMTO 700 PLUS, FEMTO, São Paulo, São Paulo, Brazil) (ë = 760 nm). Gallic acid (Sigma-Aldrich, St. Louis, MO, USA) was used as a standard at concentrations of 5–1000 µg/mL. The results are expressed in milligrams of gallic acid per gram of dry weight of extract.

### 2.6. Determination of Flavonoids

The flavonoids were determined using 1000 µL of 2% AlCl_3_ methanol solution, which was added to 1000 µL of the extracts (1000 µg/mL). After 15 min of incubation, the absorbance of the final mixture was measured using a spectrophotometer (FEMTO 700 PLUS) (ë = 430 nm) [40]. Rutin (Sigma-Aldrich, St. Louis, MO, USA) was used as a standard at concentrations of 1–50 µg/mL. The flavonoid contents are expressed in milligrams per gram of dry weight of extract.

### 2.7. Determination of Condensed Tannin

For the condensed tannin test, samples (1000 µg/mL) were mixed with 5 mL of vanillin-HCl (8% aq. conc. HCl and 4% vanillin in methanol). Methanol served as the blank, and a standard curve of catechin (Sigma-Aldrich, St. Louis, MO, USA) was used. The mixture was incubated in a water bath for 20 min, and then, the absorbance was measured at 510 nm [41]. The results are expressed in catechin milligrams per gram of dry weight of extract.

### 2.8. Determination of Alkaloids

The total alkaloid contents in the samples were quantified according to the procedure developed by [42]. In the analysis, 40 mL of extract at a 1000-µg/mL concentration was used and acidified to pH 2–2.5 with 1-N HCl and 4 mL of Dragendorff reagent and centrifuged at 2400 rpm for 30 min. The supernatant was discarded, and the residue was treated with 1 mL of solute ethyl alcohol; 2 mL of 1% sodium sulfite was added, and the mixture was centrifuged at 2400 rpm for 30 min. The supernatant was then discarded, and the residue was treated with 2 mL of concentrated nitric acid; the resulting contents were transferred to a 50-mL volumetric flask and brought to volume with distilled water. Then, 1 mL of this solution was taken, and 5 mL of 3% (w/v) thiourea was added; the mixture of nitric acid and thiourea was used as a blank, and the sample’s absorbance read at 435 nm was measured. Berberine (Sigma-Aldrich, St. Louis, MO, USA) was employed as the standard, and linearity was obtained between 40 and 200µg/mL. The alkaloid contents are expressed in milligrams per gram of dry weight of extract.

### 2.9. Determination of Antioxidant Activity

The antioxidant activity of the extracts was assessed using the free radical indicator DPPH (1,1-diphenyl-2-picrilhidrazyl) (Sigma-Aldrich, St. Louis, MO, USA) [43]. The percentage of inhibition by each concentration was used to obtain the IC_50_ values, which were the minimum concentrations of antioxidant necessary to reduce the initial concentration of DPPH by 50%. The experiment was carried out in a room under a light shelter, with a controlled temperature (25 ± 1 °C). To calculate the minimum inhibitory concentrations (IC_50_), the extracts were prepared with distilled water at the following concentrations: 5, 10, 20, 200, 30, 40, 50, 60, 70, 80, 90 and 100 µg/mL. Based on the sequestering activity of the different dilutions of the sample, a graph was plotted with the % reduction of DPPH on the Y-axis and the concentration of the extracts (µg/mL) on the X-axis to determine the concentration of the sample necessary to reduce 50% of the DPPH and a correlation coefficient. The coefficients (r) obtained for the samples were 0.989 for *L. tomentosa*, 0.991 for *L. longifolia*, 0.990 for *L. sericea* and 0.986 for *L. nervosa*.

### 2.10. Data Analyses

The experiment was entirely randomized, with five treatments (four plants and one control), each replicated ten times, with five subsamples and a total of 50 larvae per treatment. The reproductive stage was studied with 10 repetitions, each represented by a cage containing a couple of *P. xylostella*. Data normality was assessed by the Shapiro–Wilk test. The results were subjected to the Kruskal–Wallis test (*p* ≤ 0.05). All analyses (2.5, 2.6, 2.7, 2.8 and 2.9) were performed in triplicate, and the results are expressed as mean ± confidence interval (95%). The data were analyzed using the R platform, and *p*-values lower than 0.05 (*p* < 0.05) were considered as indicative of significant differences between the samples compared in each test.

## 3. Results

The aqueous *Ludwigia* extracts prolonged the larval phase, especially for the *L. longifolia* extract (χ^2^ = 16.801; df = 4; *p* = 0.0021) (Table 1). The percentages of larval survival were lower when all the extracts were used. However, *L. tomentosa* caused a higher mortality in *P. xylostella* larvae (χ^2^ = 10.100; *p* = 0.0387) (Table 1). Extracts from *Ludwigia* species prolonged the pupal duration (χ^2^ = 9.8882; *p* = 0.0423), pupal survival (χ^2^ = 562; *p* = 0.2176) and increased the sex ratio (χ^2^ = 0.2849; df = 4, *p* = 0.9908) of *P. xylostella* (Table 1). We observed that the pupal weights of the larvae treated with *L. longifolia* (3.87 g) and *L. tomentosa* (4.58 g) were significantly reduced when compared to the control (5.38 g) (χ^2^ = 21.202; *p* = 0.0002) (Table 1).

**Figure 3:**
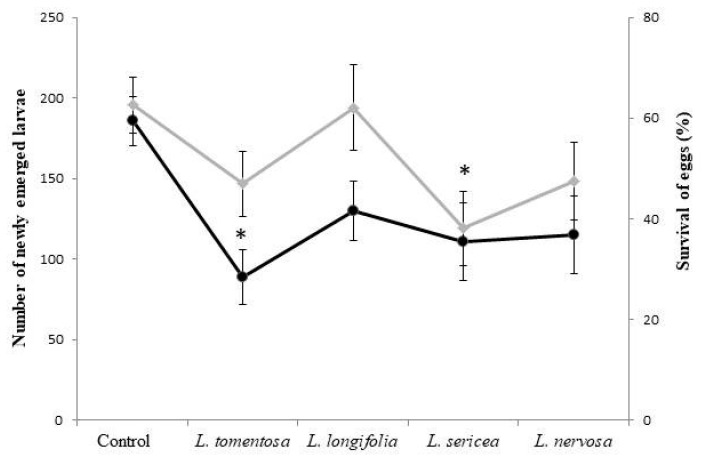
Number of newly emerged larvae (dark line) and survival of eggs (%) (light line) of *Plutella xylostella* treated with *Ludwigia* spp. aqueous extracts. Each data point represents mean ± standard error. (●) Mean number of newly emerged larvae, and (♦) mean percentage of survival of eggs. Asterisks indicate significant differences (* p < 0.05), as determined by the Kruskal–Wallis test.

In the adult stage of *P. xylostella*, the extracts did not significantly influence the longevity of the males (÷2 = 3.5399; *p* = 0.4718) or females (χ^2^ = 6.6539; *p* = 0.1553) or the oviposition period (χ^2^ = 3.2754; *p* = 0.5128) (Table 2). We observed a reduction in fertility (÷2 = 19.846; *p* = 0.0005) (Table 2) and the number of newly emerged *P. xylostella* larvae (χ^2^ = 11.421; *p* = 0.0222) (Figure 3) of *P. xylostella* for all the treatments. We also noticed a significant reduction with the *L. tomentosa* extract. Egg survival was reduced by the *L. sericea* extract (Figure 3).

The antioxidant activity was more prominent for *L. longifolia*, *L. sericea*, *L. tomentosa* and *L. nervosa*, in that order. Phenolic compounds, flavonoids, condensed tannins and alkaloids were found in greater quantities in the extracts of *L. nervosa*, *L. tomentosa*, *L. sericea* and *L. longifolia*, in that order (Table 3).

## 4. Discussion

The antibiosis effect of *Ludwigia* extracts was evidenced by the mortality of the individuals in the larval phase and the reduced pupal weight, fecundity, number of newly emerged larvae and percentage of surviving eggs. These phenomena can occur when interfering substances inhibit the food consumption during morphological and physiological transformations, which require intense biochemical activity [44,45,46,47]. The phytochemical screening showed that the *Ludwigia* species presented all the classes of compounds studied. Our results could be attributed to some of these classes. The prolongation of the larval stage observed during the treatments with the *Ludwigia* extracts could be due to substances that hinder feeding. These can extend the larval stage due to a lower conversion of ingested aliments [48]. This can occasionally lead to the larvae’s death, principally when using the *L. tomentosa* extract, as we observed. It could also have caused the reduction in the weights of the pupae when the larvae were treated with the *L. tomentosa* and *L. longifolia* extracts.

Studies on herbivores have shown their ability to block leaf consumption, inhibit digestion and, also, create free radicals. The last can rupture the membrane and cause disorders inside insects’ intestinal systems [49], as well as delaying pupal development [50], as observed for all the extracts studied.

Flavonoids are responsible for the reduced growth of larvae [51] and pupal survival [52], impaired feeding, digestion inhibition and the release of free radicals [53]. Flavonoids such as quercetin 3-arabinoside, quercetin 3-glucoside and quercetin 3-rutinoside have already been identified in some species of *Ludwigia* [54] and found to be able to act as phagodeterrents, depending on the concentrations used [35].

Tannins are another class of compounds with anti-alimental effects [55]. The alkaloids observed may also interfere with neuroendocrine control by inactivating acetylcholinesterase in larvae, causing neurotoxicity [56], in addition to a decrease in weight and increased mortality. This is indicated by a significant decrease in proteins, glycogen, lipids and the activity of the digestive enzyme α-amylase [36].

By interfering with the larvae’s alimentation (without causing death), the compounds can influence the number of ovaries and, therefore, reduce egg production [57]. Studies have shown the biological impact of the flavonoid rutin on the fertility and survival of *P. xylostella* eggs [22], as well as that of the alkaloid piperine in *Spodoptera frugiperda* (J.E. Smith) (Lepidoptera: Noctuidae) eggs [58]. This same alkaloid has been found in *L. hyssopifolia* (G. Don) Exell [59]. The presence of both flavonoids and alkaloids can explain some of the results concerning fertility, the number of hatched eggs and the amount of eggs that survived from the *L. tomentosa* and *L. sericea* extracts.

When analyzing each species’ phenolic compounds, flavonoids, condensed tannins, alkaloids and antioxidant activity, statistically significant differences were found (*p* < 0.05). The species that showed bioactivity in this study were not those that exhibited higher amounts of phenolic compounds, flavonoids, alkaloids and condensed tannins. *L. sericea*, *L. longifolia* and *L. tomentosa* showed greater antioxidant activity, which may be directly linked to the presence of certain flavonoids such as quercetin [60]. However, it is still necessary to keep studying these cases to understand the synergic interactions between compounds.

Among the *Ludwigia* species presented here, the phytochemical screening of compounds allowed us to use a *L. nervosa* with a greater quantity of phenolic compounds, flavonoids, tannins and alkaloids. However, the use of the aqueous extract of this plant in *P. xylostella* did not reduce the number of insects in the tests we performed. Some ideas are raised and should be further studied, such as the use of other solvents, the compounds present and the use of other parts of the plant (stem bark and leaves)—factors that could influence the results obtained [61]. Such findings are well-described in the scientific literature, with studies showing that the aqueous extract of the stem bark of *Stryphnodendron adstringens* (Mart.) Coville at 10% did not affect the oviposition of *P. xylostella* [62]. However, the aqueous extract of the leaf at the same concentration inhibited female oviposition [63]. In another study, the methanolic extract of the leaves and stem bark of *S. adstringens* suppressed oviposition at all tested concentrations, causing a decrease in fertility and the number of newly emerged larvae [28].

## 5. Conclusions

*L. tomentosa*, *L. longifolia* and *L. sericea* are bioactive species, with *L. tomentosa* standing out among them. These species showed the best results regarding their ability to control *P. xylostella* populations, as they influenced important biological characteristics of these insects. Besides that, phytochemical screening showed that the aqueous extracts from these plants contained phenolic compounds, flavonoids, condensed tannins and alkaloids, substances that are able to inhibit food consumption and interfere with the morphological and physiological transformations of the offspring and oviposition of adults. Thus, *L. tomentosa*, *L. longifolia* and *L. sericea* extracts have great potential for use in the control of this pest.

## Figures and Tables

**Table 1:** Duration (days) of and survival (%) during larval and pupal stages, pupal weight (mg) and sex ratio of *Plutella.* *xylostella* L. treated with *Ludwigia* spp. aqueous extracts (25 ± 2 °C; 55 ± 5% relative humidity; 12-h photo phase).

Treatments	Larval Phase		Pupal Phase			
	Larval Duration (Days)	Larval Survival (%)	Pupal Duration (Days)	Pupal Survival (%)	Pupal Weight (Mg)	Sex Ratio
Control	6.54 ± 0.2 b	88.00 ± 4.4 a	5.95 ± 0.1 a	93.50 ± 3.3 a	5.38 ± 0.1 a	0.38 ± 0.1 a
	n = 50	n = 50	n = 44	n = 44	n = 44	n = 41
*L. tomentosa*	6.76 ± 0.3 ab	62.00 ± 7.0 b	6.47 ± 0.2 a	88.83 ± 4.7 a	4.58 ± 0.1 bc	0.43 ± 0.1 a
	n = 50	n = 50	n = 31	n = 31	n = 31	n = 27
*L. longifolia*	7.72 ± 0.2 a	72.00 ± 6.8 ab	6.42 ± 0.1 a	77.30 ± 8.4 a	3.87 ± 0.3 c	0.42 ± 0.1 a
	n = 50	n = 50	n = 35	n = 35	n = 35	n = 27
*L. sericea*	6.56 ± 0.1 b	80.00 ± 6.0 ab	6.25 ± 0.1 a	97.50 ± 2.5 a	4.92 ± 0.1 ab	0.39 ± 0.1 a
	n = 50	n = 50	n = 40	n = 40	n = 40	n = 39
*L. nervosa*	7.34 ± 0.3 ab	86.00 ± 5.2 ab	6.01±0.1 a	86.66 ± 4.9 a	5.11 ± 0.2 ab	0.45 ± 0.1 a
	n = 50	n = 50	n = 43	n = 43	n = 43	n = 37
CV (%)	11.4	24.8	7.2	18.8	13.0	79.4

Means followed by different letters in the same column differ at the 5% significance level when compared using the Tukey test; n = number of individuals. CV—coefficient of variation.

**Table 2:** Longevity of males and females (days), oviposition (days), fecundity and number of newly emerged larvae of *Plutella xylostella* treated with *Ludwigia* spp. aqueous extracts (25 ± 2 °C; 55 ± 5% relative humidity; 12-h photo phase).

Treatments	Longevity of Males (Days)	Longevity of Females (Days)	Oviposition (Days)	Fecundity	Number of Newly Emerged Larvae
Control	19.10 ± 1.8 a	18.60 ± 1.5 a	13.90 ± 0.8 a	299.40 ± 13.8 a	185.60 ± 15.0 a
	n = 10	n = 10	n = 10	n = 10	n = 10
*L. tomentosa*	21.70 ± 2.0 a	13.20 ± 1.5 a	11.30 ± 1.5 a	184.40 ± 20.3 b	88.60 ± 17.0 b
	n=10	n = 10	n = 10	n = 10	n = 10
*L. longifolia*	22.20 ± 2.1 a	18.40 ± 1.7 a	13.40 ± 1.0 a	210.90 ± 10.0 ab	129.80 ± 18.3 ab
	n = 10	n = 10	n = 10	n = 10	n = 10
*L. sericea*	22.70 ± 1.8 a	16.30 ± 1.0 a	12.30 ± 1.1 a	297.70 ± 29.3 a	110. 90 ± 24.2 ab
	n = 10	n = 10	n = 10	n = 10	n = 10
*L. nervosa*	17.50 ± 1.9 a	17.70 ± 1.7 a	13.70 ± 1.5 a	229.50 ± 31.6 ab	115.40 ± 24.1 ab
	n = 10	n = 10	n = 10	n = 10	n = 10
CV (%)	26.4	27.1	29.3	29.4	51.0

Means followed by different letters in the same column differ at the 5% significance level when compared using the Tukey test; n = number of individuals. CV—coefficient of variation.

**Table 3:** Antioxidant activity data (IC_50_—minimum inhibitory concentration), phenolic compounds, flavonoids, condensed tannins and alkaloids of species of *Ludwigia*.

Aqueous Extract	Antioxidant Activity IC_50_ (μg/ mL)	Phenolic Compounds (mg/g)	Flavonoids (mg/g)	Condensed Tannins (mg/g)	Alkaloids (mg/g)
*Ludwigia tomentosa*	12.8 ± 0.3	299.4 ± 0.7	162.3 ± 0.9	32.5 ± 0.2	11.2 ± 0.1
*Ludwigia longifolia*	14.9 ± 0.4	289.7 ± 0.8	144.9 ± 0.8	30.9 ± 0.1	10.4 ± 0.1
*Ludwigia sericea*	13.5 ± 0.2	291.5 ± 1.2	153.7 ± 1.5	31.8 ± 0.1	10.7 ± 0.1
*Ludwigia nervosa*	9.6 ± 0.1	312.4 ± 0.9	188.8 ± 1.3	34.3 ± 0.2	12.5 ± 0.2
